# Table Manners

**Published:** 1878-09

**Authors:** 


### TABLE MANNERS.

OF all the articles copied from the JOURNAL OF HEALTH, that has had the widest and most persistent circulation, which suggested the importance of the family meeting around the dinner-table, and breakfast and supper as well, in a happy, loving, and cheerful frame of mind. It went across the water, and returned as copied from *Chambers's Journal*, under which credit it is going the rounds of the American press again. It was scarcely half a page, but it has caused us a great deal of reflection. Why was it so extensively copied at home and abroad? Was it because of the justness of its views? or rather that the experience of the press, as to the reverse of the picture, is so universal? If so, many gentlemen of education and culture have experienced the sad feeling of having wives or children come to the table, only to fret, and growl, and complain, and sulk. It is horrible to think of. And yet it may be presumed, that the happiness of quite as many excellent wives is marred, if not wholly eaten out, by husbands who come to the table with a terrible dignity! or with a selfishness so predominant that it places every body else and every thing under tribute to its supreme gratification; moroseness stamped on every feature; a belittling querulousness in every uttered sentence. Here one comes now as stately as a turkey-cock, as cross as a bear, and as rough as a corn-cob. He speaks in short, crusty words; the innocent prattle of his children is an apparent torture to him; there must not be a whimper or a whisper, for he is poring over a newspaper, or in the midst of some plan or project for gain or fame. His very presence is felt as a cloud, an incubus, an iceberg; and there is only gladness when he is gone; it is then only that the sunshine of family affection and love comes out, and filial and motherly sympathies well up from loving hearts.

To meet at the breakfast-table, father, mother, children, all well, ought to be a happiness to any heart; it should be a source of humble gratitude, and should wake up the warmest feelings of our nature. Shame upon the contemptible and low-bred cur, whether parent or child, that can ever come to the breakfast-table, where all the family have met in health, only to frown, and whine, and growl, and fret! it is *prima facie* evidence of a mean and groveling and selfish and degraded nature, whencesoever the churl may have sprung. Nor is it less reprehensible to make such exhibitions at the tea-table; for before the morning comes, some of the little circle may be stricken with some deadly disease, to gather around that table not again forever! Children in good health, if left to themselves at the table, become, after a few mouthfuls, garrulous and noisy; but if within at all reasonable or bearable bounds, it is better to let them alone; they eat less, because they do not eat so rapidly as if compelled to keep silent, while the very exhilaration of spirits quickens the circulation of the vital fluids, and energizes digestion and assimilation. The extremes of society curiously meet in this regard. The tables of the rich and the nobles of England are models of mirth, wit, and bonhommie; it takes hours to get through a repast, and they live long. If any body will look in upon the negroes of a well-to-do family in Kentucky, while at their meals, they can not but be impressed with the perfect *abandon* of jabber, cachinnation, and mirth; it seems as if they could talk all day, and they live long. It follows, then, that at the family-table all should meet, and do it habitually, to make a common interchange of high-bred courtesies, of warm affections, of cheering mirthfulness, and that generosity of nature, which lifts us above the brutes which perish, promotive as these things are of good digestion, high health, and a long life.

